# Influence of flow rate, fluid temperature, and extension line on Hotline and S-line heating capability: an in vitro study

**DOI:** 10.1186/s12871-020-01225-1

**Published:** 2021-01-04

**Authors:** Hosu Kim, Tae Kyong Kim, Sukha Yoo, Jin-Tae Kim

**Affiliations:** 1 College of Medicine, Seoul National University, Seoul, Republic of Korea; 2 Department of Anesthesiology and Pain Medicine, SMG-SNU Boramae Medical Center, Seoul, Republic of Korea; 3 Department of Anesthesiology and Pain Medicine, Seoul National University Hospital, 101 Daehak-ro, Jongno-gu, 03080 Seoul, Republic of Korea

**Keywords:** Hypothermia, Barkey S-line, Flow rate, Extension line, Fluid temperature, Heating capability

## Abstract

**Background:**

A fluid warmer can prevent hypothermia during the perioperative period. This study evaluated the heating capabilities of Hotline and Barkey S-line under different flow rates and initial fluid temperatures, as well as after the extension line installation.

**Methods:**

We measured the temperature of a 0.9% sodium chloride solution at the fluid warmer outlet (TProx) and the extension line end (TDistal) with three different initial fluid temperatures (room, warm, and cold) and two flow rates (250 ml/hr and 100 mL/hr).

**Results:**

At a 250 ml/hr flow rate, the TProx and TDistal values were observed to be higher in Hotline than in S-line when using room-temperature or cold fluid. Administering of the warm fluid at the same flow rate significantly increased the TProx and TDistal values in S-line more than the cold and room-temperature fluids. At flow rates of 100 ml/hr, TDistal values were significantly lower than TProx values in both devices regardless of the initial fluid temperature.

**Conclusions:**

Hotline outperformed S-line for warming fluids at a high flow rate with cold or room-temperature fluids. Administering warm fluid in S-line prevented a decrease in the fluid temperature at a high flow rate. However, at a low flow rate, the fluid temperature significantly decreased in both devices after passing through an extension line.

## Background

Inadvertent perioperative hypothermia commonly occurs in patients undergoing surgery on account of a cold operating room, anesthetic agents that weaken thermoregulatory control, and administration of un-warmed fluid [1–8]. Even mild hypothermia, defined as a core body temperature ranging from 34 to 36 °C, is associated with complications, such as an increased need for a blood transfusion, increased length of hospitalization, a higher incidence of postoperative myocardial infarction, and the risk of developing a surgical wound infection [7–10]. Preventing perioperative hypothermia is therefore critical.

It is recommended that physicians assess the risk factors associated with perioperative hypothermia to reduce hypothermia-related complications [3, 11]. After assessing these risk factors, physicians should employ interventions that are appropriately designed for the specific patient population and type of operation [1–3, 10]. The use of cold intravenous fluids is one of the most potentially modifiable risk factors [5, 12]. The National Institute for Health and Care Excellence (NICE) published clinical guidelines in 2008 that support the use of intravenous fluid warmers to prevent perioperative hypothermia [13]. Using such a device to warm intravenous fluids before administering it to the patient has been shown to prevent inadvertent hypothermia [4, 5, 12]. However, there are various conditions during fluid administration that must be considered, such as the fluid warmer type, flow rate, fluid temperature, and the IV line length, because the conditions may affect the actual temperature of the administered fluid. Therefore, it is critical to investigate the performance of a fluid warmer in different circumstances.

Numerous commercially available fluid warmers, such as Ranger, ThermoSens, Mega Acer Kit, FT800, and Hotline HL-90, have been investigated under different flow rates and room temperatures [12, 14–16]. However, previous studies mainly focused on changing the flow rate [12, 14, 15]. To determine the clinical effectiveness, various clinical factors such as the initial fluid temperature and the use of extension lines should also be considered.

The purpose of this study was to evaluate the fluid heating capabilities of Barkey S-line and Hotline HL-90 according to different flow rates, fluid temperatures, and the presence of an extension line.

## Methods

This study was performed in the designated spot next to the nursing station in the post-anesthesia care unit (PACU). The PACU room temperature was maintained at 23 °C, and the humidity level was maintained at approximately 20%. Normal saline fluid (0.9% sodium chloride solution, CJ, Seoul, Republic of Korea) was used for all the experiments in this study. The fluid temperature was measured using a two-channel thermometer (ThermaQ; ThermoWorks, London, UK). For Barkey S-line (Barkey GmbH & Co. KG, Leopoldshöhe, Germany), the operating temperature was maintained at 39.5 °C throughout the experiments. A standard infusion set was inserted in the 1.5-m heating profile of S-line according to the manufacturer instructions. For Hotline HL-90 (Smiths Medical, Minneapolis, MN), the operating temperature was set to 40 °C. The REF L-70 disposable tubing system (length 2.4 m, volume load 20 ml; Level 1 Technologies Inc., MA, USA) was installed according to the manufacturer instructions.

**Fig. 1:**
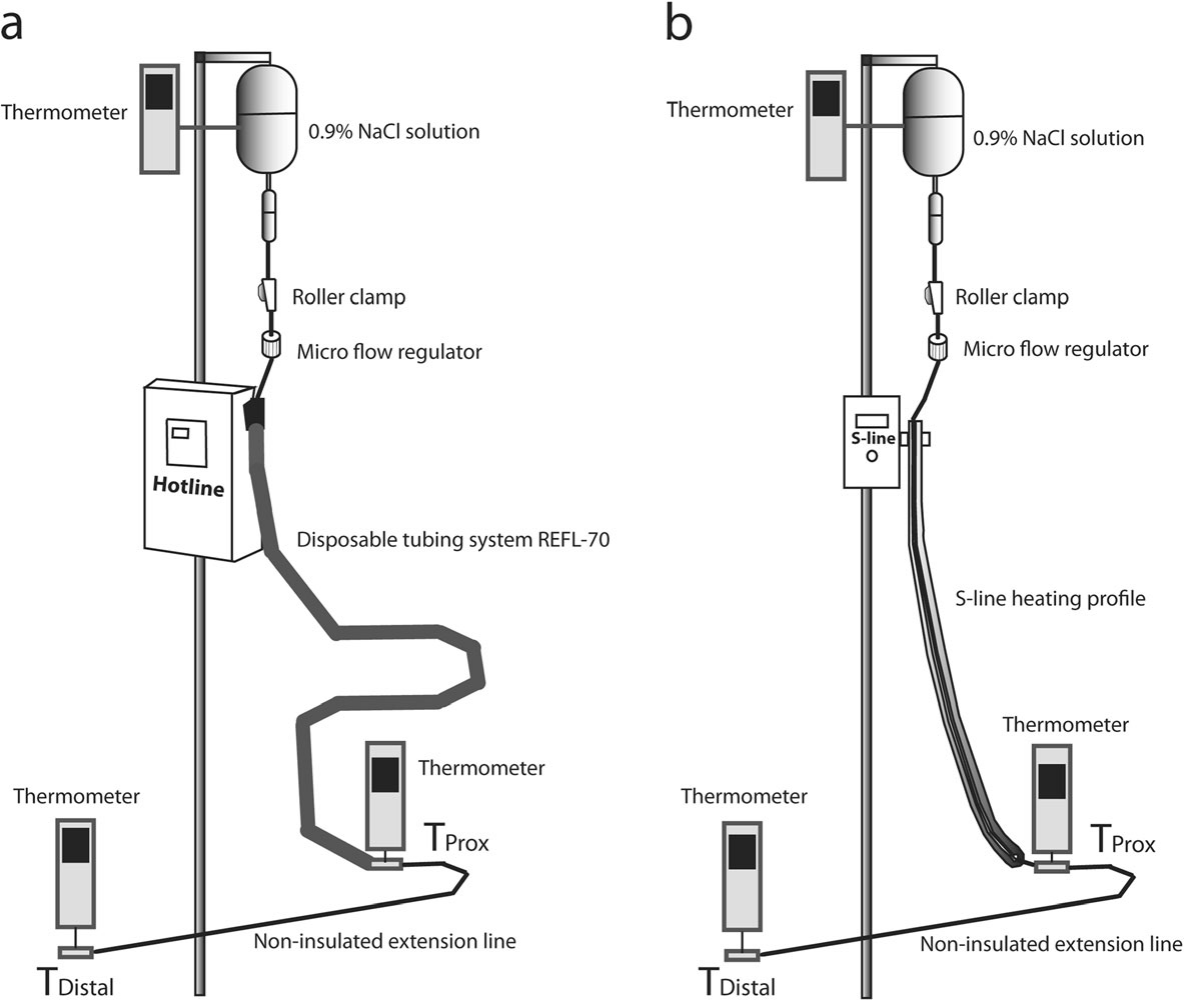
The figure shows an overview of the experimental setup. 0.9% NaCl solution is warmed as it travels down the fluid warmer. After fluid exits the warmer, it goes further down through the non-insulated extension line. The temperatures at the outlet of the warmer (T_Prox_) and the outlet of the extension line (T_Distal_) were measured using thermometers. *a* and *b* in the left and right show the setups for Hotline and S-line, respectively

We evaluated the efficacy of the two fluid warmers under several conditions. Three different conditions were set for the initial fluid temperature: room temperature (22–23 °C), cold (9–10 °C), and warm (46–47 °C). The normal saline solutions were respectively maintained for 12 h in the operating room, in a refrigerator, and in a heating cabinet for each of the three different conditions. Two distinct flow rates were tested in this study: 100 ml/hr and 250 ml/hr. The roller clamp was fully open, and the flow rate was adjusted using a micro-flow regulator (I.V. Flow Control Line, Insung Medical Co., Seoul, Republic of Korea) attached to the infusion set at a flow rate of 100 or 250 ml/hr. The regulator tolerance ranged from − 10–20% according to the manufacturer instructions. The height of the infusion bag was the same in all experiments (1.8 m). One unit of a non-insulated extension set (DEHP-free Extension Plus, SBD Medical, Republic of Korea), which was 90 cm in length, was connected to the fluid warmer outlet. The fluid temperature was measured at two points, first at the fluid warmer outlet (T_Prox_) and then at the end of the non-insulated extension line (T_Distal_). After starting each trial, the temperatures at the two positions (T_Prox_ and T_Distal_) were recorded every minute until the end of the trial (Fig. 1).

T_Prox_ and T_Distal_ were carefully evaluated for the presence of a plateau in each trial. The plateau in each trial was defined as the time point at which the measured temperatures were the most stable without a noted fluctuation for 3 min. In each trial, only the T_Prox_ and T_Distal_ values from the middle time point of the plateau were used for the subsequent statistical analysis. Five trials were performed for an individual experimental condition to obtain five data points for T_Prox_ and T_Distal_, respectively.

Statistical analysis was performed using SPSS v.20.0 for Windows (IBM SPSS, Inc., Armonk, NY) and R software version 3.4.4 (R Foundation for Statistical Computing, Austria). A data chart was produced using Microsoft Excel 2007. All fluid temperatures were reported as a median (interquartile [IQR] range). The Wilcoxon signed-rank test was used to test for a statistical difference between T_Prox_ and T_Distal_ values. The Hodges–Lehmann estimator was utilized to create the 95% confidence interval for the median difference between T_Prox_ and T_Distal_. The Mann–Whitney U test was employed to compare T_Prox_ or T_Distal_ values under different flow rates using the same fluid warmer or between Hotline and S-line at the same flow rate. The bootstrap method was applied to form the 95% confidence interval for the analyses performed using the Mann–Whitney U test. Statistical significance was defined as a *p*-value < 0.05.

## Results

### Room‐temperature fluid

**Fig. 2:**
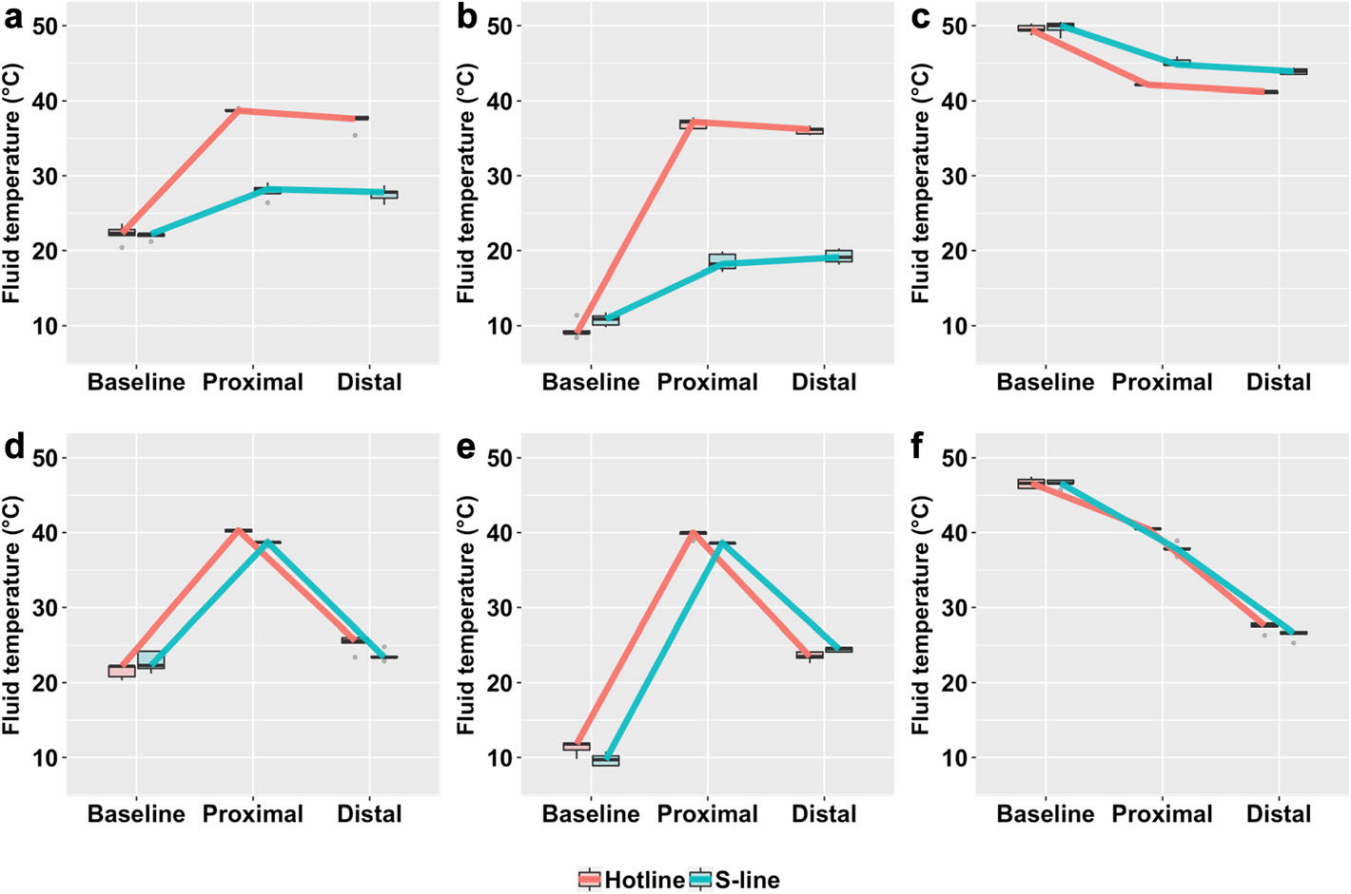
The figure illustrates the measured fluid temperatures using boxplots and line graphs. The red line and blue line connect the data from Hotline and S-line respectively. Boxes indicate the interquartile range, the bold horizontal lines in the box are the median values, and the whiskers are the range. Outliers are displayed as dots. **a** room-temperature fluid at 250 ml/hr, **b** cold fluid at 250 ml/hr, **c** warm fluid at 250 ml/hr, **d** room-temperature fluid at 100 ml/hr, **e** cold fluid at 100 ml/hr, **f** warm fluid at 100 ml/hr

The room-temperature fluid was maintained at 21–23 °C. T_Prox_ was higher in Hotline (38.7 [38.7–38.8]°C) than in S-line (28.2 [27.6–28.4]°C) based on the median difference of 10.5 °C (95% CI 9.6–12.3) at the flow rate of 250 ml/hr. At the flow rate of 100 ml/hr, the T_Prox_ value was higher in Hotline (40.3 [40.1–40.4]°C) than S-line (38.7 [38.6–38.7]°C) according to the median difference of 1.6 °C (95% CI 1.4–2.1. T_Distal_ measured higher in Hotline (37.6 [37.6–37.9]°C) than in S-line (27.8 [27.0-27.9]°C) based on the median difference of 9.8 °C (95% CI 7.6–11.5) at the flow rate of 250 ml/hr. However, the T_Distal_ values were not significantly different between Hotline (25.6 [25.3–26.0]°C) and S-line (23.4 [23.4–23.5]°C) at 100 ml/hr (Fig. 2).

T_Prox_ in Hotline was slightly higher at 100 ml/hr than at 250 ml/hr based on the median difference of 1.6 °C (95% CI 1.3–1.9). However, T_Prox_ in S-line was much higher at 100 ml/hr than at 250 ml/hr based on the median difference of 10.5 °C (95% CI 9.6–12.3).

Furthermore, T_Distal_ in Hotline was higher at 250 ml/hr than at 100 ml/hr by 12.0 °C (95% CI 9.8–14.2). T_Distal_ in S-line was higher at 250 ml/hr than at 100 ml/hr by 4.4 °C (95% CI 2.6–5.3). The T_Distal_ values were lower at 100 ml/hr than at 250 ml/hr. In addition, T_Prox_ was higher than T_Distal_ in both devices regardless of the flow rate. However, the differences were more substantial at 100 ml/hr. At a flow rate of 250 ml/hr, the median differences between T_Prox_ and T_Distal_ were 1.1 °C (95% CI 0.9–2.2) in Hotline and 0.4 °C (95% CI 0.3–0.6) in S-line. At 100 ml/hr, the median differences were 14.7 °C (95% CI 14.3–16.7) in Hotline and 15.1 °C (95% CI 13.9–16.0) in S-line.

### Cold fluid

The initial temperature of the starting solution was maintained at 9–11 °C in this group. T_Prox_ was higher in Hotline (37.2 [36.3–37.4]°C) than in S-line (18.2 [17.6–19.5]°C) by the median difference of 19.0 °C (95% CI 16.7–20.2) at 250 ml/hr. However, the difference between Hotline (40.0 [39.8–40.0]°C) and S-line (38.6 [38.5–38.6]°C) was 1.4 °C (95% CI 0.3–4.3) at 100 ml/hr. T_Distal_ at 250 ml/hr was also higher in Hotline (36.2 [35.6–36.3]°C) than in S-line (19.1 [18.5–20.0]°C) based on the median difference of 17.1 °C (95% CI 15.4–18.2). However, the T_Distal_ values from Hotline (23.5 [23.3–24.1]°C) and S-line (24.5 [24.1–24.7]°C) were not statistically different at 100 ml/hr (Fig. 2).

T_Prox_ measured in Hotline was slightly higher at 100 ml/hr than at 250 ml/hr by 2.8 °C (95% CI 1.7–3.8). In S-line, the T_Prox_ value was significantly higher at 100 ml/hr than at 250 ml/hr by 20.4 °C (95% CI 17.5–21.4). For T_Distal_ in Hotline, the temperature at 250 ml/hr was higher than at 100 ml/hr, and the median difference was 12.7 °C (95% CI 11.4–13.6). In contrast, T_Distal_ at 100 ml/hr was higher than at 250 ml/hr in S-line, and the median difference was 5.4 °C (95% CI 4.0–6.4). In both devices, the T_Distal_ values at 100 ml/hr were similar to the ambient temperature. The T_Prox_ was higher than T_Distal_ in both devices regardless of the flow rate. The only exception was observed in S-line at 250 ml/hr; T_Prox_ is 18.2 °C and T_Distal_ is 19.1 °C. The difference between T_Prox_ and T_Distal_ was greater at 100 ml/hr than at 250 ml/hr. At 250 ml/hr, the median differences between T_Prox_ and T_Distal_ were 0.9 °C (95% CI 0.6–1.2) in Hotline and 0.7 °C (95% CI 0.5–0.9) in S-line. At 100 ml/hr, the median differences were 16.3 °C (95% CI 15.8–16.6) in Hotline and 13.9 °C (95% CI 11.2–14.6) in S-line. At 100 ml/hr, the extension line reversed most of the warming and substantially cooled the fluid as it traveled from T_Prox_ to T_Distal_.

### Warm fluid

The initial fluid temperature was maintained at 46–49 °C in the warm temperature group. In this temperature group, the temperature of the initial fluid was higher than the operating temperatures of the fluid warmers themselves. The T_Prox_ value at 250 ml/hr in S-line (44.8 [44.7–45.4]°C) was higher than in Hotline (42.1 [42.1–42.1]°C) according to the median difference of 2.7 °C (95% CI 2.4–3.8). At 100 ml/hr, the T_Prox_ value was higher in Hotline (40.5 [40.5–40.5]°C) than S-line (37.8 [37.8–37.9]°C) according to the median difference of 2.7 °C (95% CI 1.6–3.7). T_Distal_ in S-line (43.9 [43.5–44.2]°C) at 250 ml/hr was higher than in Hotline (41.2 [41.0-41.2]°C) according to the median difference of 2.7 °C (95% CI 2.2–3.2). At 100 ml/hr, no statistical difference existed between the T_Distal_ values in Hotline (27.6 [27.4–27.9]°C) and S-line (26.6 [26.5–26.8]°C) (Fig. 2).

In Hotline, the T_Prox_ value was higher at 250 ml/hr than at 100 ml/hr, and the median difference was 1.6 °C (95% CI 1.5–1.7). For the T_Distal_ value, the temperature measured at 250 ml/hr was again higher, and the median difference was 13.6 °C (95% CI 13.1–14.9). Similarly, the T_Prox_ value in S-line was higher at 250 ml/hr than at 100 ml/hr; the median difference was 7.0 °C (95% CI 5.9–8.1). For the T_Distal_ value, the measurement at 250 ml/hr was higher than at 100 ml/hr based on the median difference of 17.3 °C (95% CI 16.7–18.6). T_Prox_ was higher than T_Distal_ at the two flow rates in both Hotline and S-line. A flow rate of 100 ml/hr produced larger differences between T_Prox_ and T_Distal_. At 250 ml/hr, the median differences between T_Prox_ and T_Distal_ were 1.0 °C (95% CI 0.9–1.1) in Hotline and 1.2 °C (95% CI 0.8–1.5) in S-line. At 100 ml/hr, the median differences were 12.9 °C (95% CI 12.5–14.2) in Hotline and 11.3 °C (95% CI 11.1–12.1) in S-line. The slower flow rate correlated with a more substantial difference in the median values.

## Discussion

This study showed that Hotline was superior to S-line for warming fluids when using room-temperature or cold saline at a high flow rate. However, at a low flow rate, the fluid temperature significantly decreased in both devices after passing through an extension line.

The difference in heating capacity between the two devices was likely due to the differences in their respective heating mechanisms, i.e., a coaxial circulating water bath (Hotline) versus a dry heating profile (S-line). The dry-heating system is expected to incur greater heat losses on account of the exposed portion of the extension line in ambient temperature. Thus, the Hotline coaxial warming system is apparently more effective than S-line in preventing hypothermia during rapid fluid administration. When administrating fluid rapidly, pre-warming fluid appears to be effective in maintaining fluid temperature warm especially in S-line. However, it would be important to check the warming temperature beforehand to avoid getting too hot fluid.

At 100 ml/hr, changing the initial temperature affected the T_Prox_ less in both Hotline and S-line. The slow flow rate would have provided sufficient time for both fluid warmers to affect the fluid temperatures. However, T_distal_ of the warm fluid seem to be slightly higher than those of the room-temperature or cold fluids at 100 ml/hr in both Hotline and S-line. This suggests that using pre-warmed fluid may have some role in maintaining fluid temperature even at the low flow rate when the extension line is used. Warm fluid could have influenced T_Distal_ through heating up the tube in which the fluid traveled down, however, the exact mechanism behind this finding remains to be further investigated.

There are several studies evaluating the performance of fluid warmers in literature [4, 12, 15, 16]. The previous studies produced detailed analyses on effect of changing one or two variables on the final fluid temperature among different fluid warmers. For example, the studies were descriptive of the effect of changing the flow rate or the initial fluid temperature. The current study has a strength that it analyzed how extension line, flow rate and initial fluid temperature interact to influence the final fluid temperature and presented comprehensive and clinically relevant performance profiles of Hotline and S-line.

It is clinically important to determine whether warmed fluids can be delivered to the patient without heat loss as they pass through a non-insulated extension line. Interestingly, in this study, a statistically significant change in fluid temperature was observed after the extension line in every experimental condition regardless of the fluid warmer type, initial fluid temperature, or flow rate. The change in fluid temperature was more pronounced at the low flow rate than at the high one. The fluid lost a significant amount of heat as it traveled an additional 90 cm at a rate of 100 ml/hr. This finding warrants the utilization of additional measures for hypothermia prevention when using an extension at the low flow rate, especially when administering fluids to patients with a high risk of developing hypothermia such as neonatal and older patients [17]. Furthermore, it is essential to minimize heat loss as the fluid travels down the extension line, which can be achieved by an extension line innovation or by applying supplemental measures against hypothermia.

Several limitations of this study should be noted. In an actual clinical setting, the fluid administration rate, initial fluid temperature, and extension line length may differ from the conditions used in this study. For example, during rapid fluid resuscitation, the infusion rate can be as high as 60 to 80 mL/kg per hour [18]. Also, in this study, the fluid temperature in the warm temperature group was greater than 45 °C, which can be problematic because the proteins in red blood cells can degenerate at a temperature higher than 45 °C [19]. Moreover, precautionary measures should be taken against preparing fluids that are excessively hot for clinical practice. Finally, extension lines longer than 90 cm can be employed in certain clinical scenarios. Longer extension lines could affect the fluid temperature more markedly than the line length employed in this study.

## Conclusions

In summary, Hotline outperformed S-line for warming fluids at a high flow rate with cold or room-temperature fluids. Administering warm fluid in S-line prevented a decrease in the fluid temperature at a high flow rate. Finally, using an extension line can reduce the final fluid temperature delivered to the patient at a low flow rate in both devices.

## Data Availability

The datasets used and/or analysed during the current study are available from the corresponding author on reasonable request.

## Abbreviations

T_Prox_: Temperature at the fluid warmer point
T_Distal_: Temperature at the extension line point
PACU: Post-anesthesia care unit
